# Arthroscopic Bankart repair with an individualized capsular shift restores physiological capsular volume in patients with anterior shoulder instability

**DOI:** 10.1007/s00167-020-05952-3

**Published:** 2020-04-02

**Authors:** Helge Eberbach, Martin Jaeger, Lisa Bode, Kaywan Izadpanah, Andreas Hupperich, Peter Ogon, Norbert P. Südkamp, Dirk Maier

**Affiliations:** 1 Department of Orthopaedic and Trauma Surgery, Medical Center-University of Freiburg, Faculty of Medicine, University of Freiburg, Hugstetter Straße 55, 79106 Freiburg, Germany; 2 Center of Orthopaedic Sports Medicine, Breisacher Str. 84, 79110 Freiburg, Germany

**Keywords:** Shoulder, instability, Arthroscopic, Bankart repair, Capsular shift, Capsular volume

## Abstract

**Purpose:**

Capsular volume reduction in the context of anterior arthroscopic shoulder stabilization represents an important but uncontrolled parameter. The aim of this study was to analyse capsular volume reduction by arthroscopic Bankart repair with an individualized capsular shift in patients with and without ligamentous hyperlaxity compared to a control group.

**Methods:**

In the context of a prospective controlled study, intraoperative capsular volume measurements were performed in 32 patients with anterior shoulder instability before and after arthroscopic Bankart repair with an individualized capsular shift. The results were compared to those of a control group of 50 patients without instability. Physiological shoulder joint volumes were calculated and correlated with biometric parameters (sex, age, height, weight and BMI).

**Results:**

Patients with anterior shoulder instability showed a mean preinterventional capsular volume of 35.6 ± 10.6 mL, which was found to be significantly reduced to 19.3 ± 5.4 mL following arthroscopic Bankart repair with an individualized capsular shift (relative capsular volume reduction: 45.9 ± 21.9%; *P* < 0.01). Pre-interventional volumes were significantly greater in hyperlax than in non-hyperlax patients, while post-interventional volumes did not differ significantly. The average shoulder joint volume of the control group was 21.1 ± 7.0 mL, which was significantly correlated with sex, height and weight (*P* < 0.01). Postinterventional capsular volumes did not significantly differ from those of the controls (n.s.).

**Conclusion:**

Arthroscopic Bankart repair with an individualized capsular shift enabled the restoration of physiological capsular volume conditions in hyperlax and non-hyperlax patients with anterior shoulder instability. Current findings allow for individual adjustment and intraoperative control of capsular volume reduction to avoid over- or under correction of the shoulder joint volume. Future clinical studies should evaluate, whether individualized approaches to arthroscopic shoulder stabilization are associated with superior clinical outcome.

## Introduction

Along with structural lesions of the labrum and bony defects of the glenoid or humeral head, capsular volume enlargement represents the main pathogenetic and prognostic factor for anterior glenohumeral instability [18, 31, 35]. In particular, patients with ligamentous hyperlaxity and a pathological increase in capsular volume are at risk for recurrent instability of the shoulder [20, 26]. In event of failure of conservative treatment, arthroscopic Bankart repair with a variable extent of capsular shift represents the current gold standard for operative shoulder stabilization, provided that relevant bony defects are absent [4, 30]. Open shoulder stabilization may be indicated in selected patients with a high risk of arthroscopic failure, including those with ligament laxity, in high-risk patients even in the absence of bone loss, and in patients with failure of a previous well-performed arthroscopic repair [3].

Compared to open procedures, modern arthroscopic techniques offer major advantages, including the ability to treat concomitant intra-articular pathologies and preserve the musculotendinous unit of the subscapularis, while showing equivalent results in terms of functional outcomes and recurrence rates [2, 6, 13, 16, 38]. In addition to labral repair and re-tensioning of the inferior glenohumeral ligament (IGHL), restoration of a physiological joint volume is essential for successful stabilization of the shoulder joint. The arthroscopic capsular shift technique has proven to be an effective procedure for capsular volume reduction [21, 30]. Numerous cadaveric studies have shown significant capsular volume reduction using different arthroscopic capsular shift techniques [7, 11, 17, 23–25, 39]. However, a cadaveric model never reflects a realistic, clinical scenario of an unstable shoulder joint. Soft tissue biomechanical properties, concomitant pathologies, the age of specimens and experimental settings differ substantially from clinical scenarios involving arthroscopic capsular shift procedures [22, 39]. Furthermore, reliable normative values for physiological shoulder joint volumes are lacking thus far. Therefore, even experienced arthroscopic shoulder specialists are unable to assess the extent of capsular volume reduction achieved by arthroscopic capsular shift procedures. Overcorrection of the capsular volume might cause restriction of the range of motion and limitation of functional outcomes. Undercorrection might lead to persistent or recurrent glenohumeral instability [29, 30]. Only one in vivo study has investigated the effect of arthroscopic shoulder stabilization on capsular volume reduction intraoperatively. Lubiatkowski et al. found significant capsular volume reduction after labral repair (by an average of 37%) and an even higher capsular volume reduction after capsular plication (by an average of 61%) [21]. However, this study only analysed and compared labral repair and capsular plication as isolated procedures. Modern arthroscopic techniques consist of a Bankart (labral) repair combined with a capsular shift. The extent of capsular shift (capsular volume reduction) needs to be adjusted to individual requirements, most importantly to the extent of capsular redundancy and the presence of ligamentous hyperlaxity. To the best of our knowledge, no study has evaluated the effect of arthroscopic Bankart repair combined with an individualized capsular shift on capsular volume reduction compared to that in a conclusive control group.

The aims of this study were to (1) measure the capsular volumes of unstable shoulders of hyperlax and non-hyperlax patients compared to a control group without hyperlaxity, (2) quantify capsular volume reduction following arthroscopic Bankart repair with an individualized capsular shift and (3) investigate the influence of biometric parameters on the physiological shoulder joint volume. That shoulder joints of patients with anterior instability with and without ligamentous hyperlaxity would show higher preoperative capsular volumes than those of controls was hypothesized. Furthermore, we presumed that arthroscopic Bankart repair with an individualized capsular shift would enable restoration of physiological shoulder joint volumes.

## Materials and methods

This study was approved by the University of Freiburg (Germany) Institutional Review Board (IRB) (114/17). From 2013 to 2017, a prospective controlled study was performed at the Department for Orthopedic Surgery and Traumatology at the University Medical Center, Freiburg, to evaluate the effect of arthroscopic Bankart repair with an individualized capsular shift on the capsular volume of unstable shoulders. The inclusion criteria of the instability group were as follows: Bankart/ALPSA lesion in the context of posttraumatic anterior-inferior (unidirectional) shoulder instability with and without ligamentous hyperlaxity (type B2 and B3 according to Gerber and Nyffeler [12]) and failure of a conservative treatment. The exclusion criteria were multidirectional or posterior shoulder instability, previous shoulder surgery, more than three episodes of shoulder dislocation in the physical exam, radiological signs of anterior glenoid or humeral bone deficiency, and concomitant glenohumeral pathologies other than a Bankart lesion (e.g., humeral avulsion of the glenohumeral ligaments (HAGL), SLAP lesion, rotator cuff tear). Acute Bankart lesions (< 6 weeks) were excluded to prevent fluid escape. Ligamentous hyperlaxity was diagnosed clinically. All hyperlax patients showed a positive bilateral sulcus sign, a positive hyperabduction (Gagey) test on the healthy contralateral side and hyperextension of the elbow and metacarpophalangeal joints. These clinical signs were negative in non-hyperlax patients.

The capsular volumes of the instability group were compared to those of a control group of patients undergoing arthroscopic treatment due to refractory calcific tendinitis of the rotator cuff. In these patients, we routinely performed diagnostic glenohumeral arthroscopy to exclude concomitant intra-articular pathologies. The exclusion criteria in the control group were the presence of glenohumeral instability or ligamentous hyperlaxity, preoperative restriction of the range of shoulder motion, pathological capsular pattern (e.g., adhesive capsulitis, shoulder stiffness), rotator cuff tear and a history of previous shoulder surgeries.

### Operative technique

All surgeries were performed by four experienced arthroscopic shoulder specialists (DM, MJ, NPS, PO) employing a standardized operative technique (arthroscopic Bankart repair with an individualized capsular shift).

**Fig. 1:**
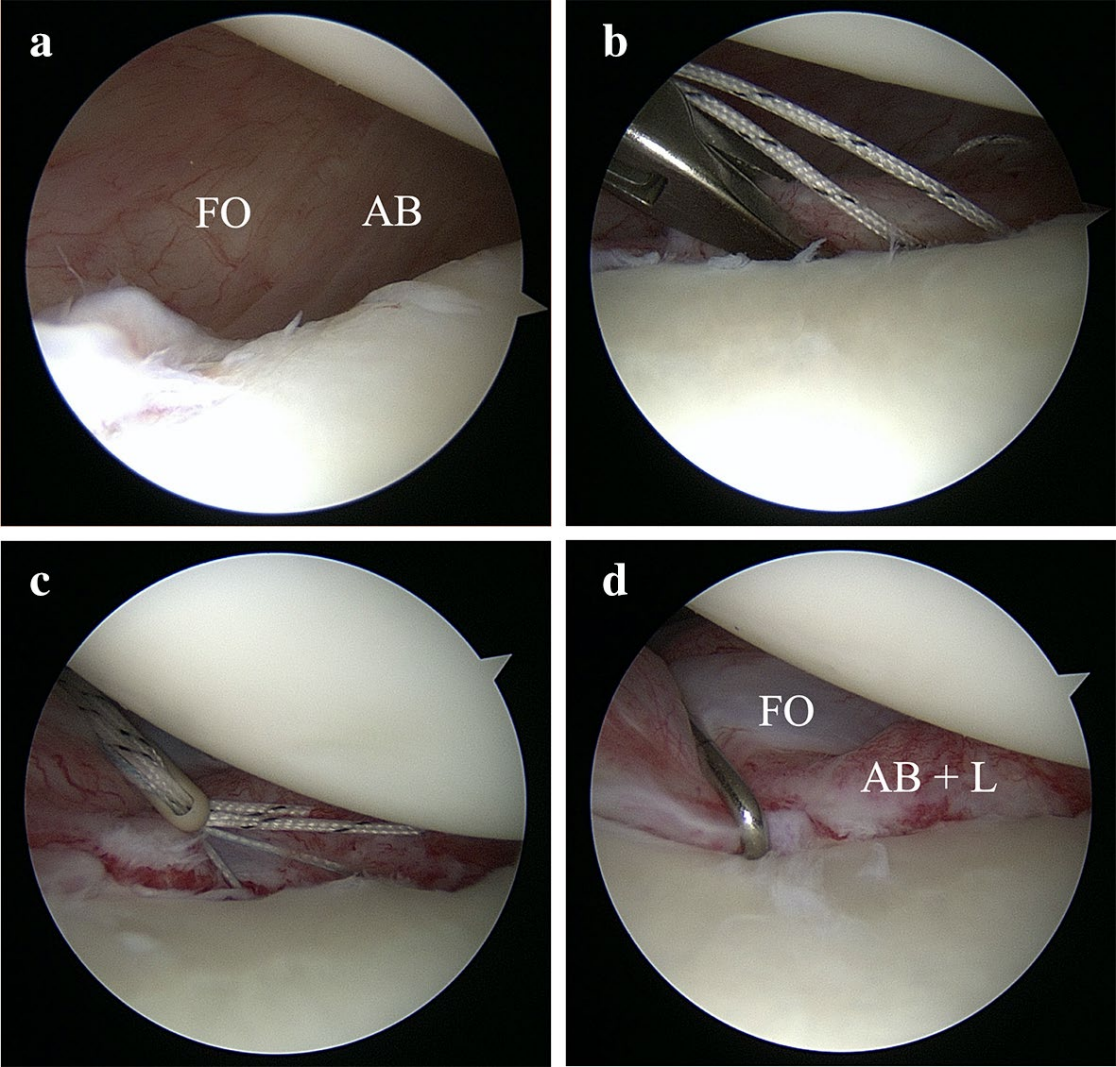
Operative Technique for Arthroscopic Bankart Repair with an Individualized Capsular Shift. Right shoulder in lateral decubitus position (**a**, view from the anterior–superior portal; **b**–**d**, view from the posterior portal). **a** Redundant anterior-inferior capsule in a hyperlax patient with an ALPSA lesion, IGHL avulsion and anterior decentring of the humeral head before arthroscopic Bankart repair with an individualized capsular shift. Both the anterior band (AB) of the inferior glenohumeral ligament and the fasciculus obliquus (FO) are unfolded, showing an oblique course to each other. **b** Simulation of physiological capsular pre-tensioning using arthroscopic forceps and determination of the individually required extent of capsular shift. **c** Caspulolabral IGHL sutures loaded into the eyelet of the suture anchor. Prior to insertion of the anterior-inferior anchor at the 5.00 position, each suture strand was selectively pre-tensioned until complete unfolding of the inferior capsular pouch was achieved. **d** Arthroscopic Bankart repair with an individualized capsular shift with anatomic, knotless anterior-inferior capsulolabral reconstruction of the fasciculus obliquus (FO), anterior band (AB) and labrum (L)

Patients were positioned in the lateral decubitus position using a 3-point shoulder distraction system (Arthrex, Naples, FL, USA). The 3-portal technique was employed as described previously [15]. Sutures and anchors were placed through the anterior standard portal. After visualization of the capsulolabral lesion (Fig. 1a), the anterior-inferior capsulolabral complex was dissected and mobilized from superiorly to inferiorly (until 6.00 p.m.). A curette was used to prepare a 2-mm broad cortical bone bed on the anterior edge of the glenoid face. Knotless 3.5-mm Bio-PushLock anchors (Arthrex, Naples, FL) were routinely used for refixation of the anterior-inferior capsulolabral complex at the 5.00 position (right shoulder) and a double-loaded 3.0-mm Bio-FASTak anchor (Arthrex, Naples, FL) for anterior refixation at the 3.30 position.

### Individualized capsulolabral repair

Individualization of the capsulolabral repair was based on fundamental anatomical studies of the glenohumeral ligaments and the fasciculus obliquus in particular [15, 32]. For individual adjustment of the required capsular shift, the redundant capsule was grasped and reduced with forceps (Fig. 1b) to simulate physiological capsular pre-tensioning. The determined perforation site was marked with an electrothermal device. In neutral rotation and slight abduction, both the anterior band (AB) of the inferior glenohumeral ligament (ABIGHL) and the fasciculus obliquus (FO) were unfolded, showing an oblique course to each other. The passing device (25° tight-curved suture lasso; Arthrex, Naples, FL) was passed through the avulsed ABIGHL while incorporating the superior edges of the FO and the labral tissue (L) at the 6.00 and 5.30 positions to create an anatomical inferior mattress stitch. For this purpose, FiberWire #0 or #2 (Arthrex, Naples, FL) were used according to local tissue quality. The same procedure was repeated at the 5.00, 4.30 and 4.00 positions to place additional stitches as needed. Four or five capsulolabral sutures were used for unfolding the enlarged inferior capsular pouch, resulting in anatomical capsulolabral refixation from the 4.00 position to the 6.00 position. All sutures were placed through the eyelet of the 3.5-mm Bio-PushLock anchor. After drilling, the suture-loaded eyelet was placed into the drill hole. Before anchor insertion, each suture strand was selectively tensioned under arthroscopic control until the capsular pouch was fully unfolded (Fig. 1c). Physiological conditions, in the case of re-creation of the labral “bump” and unfolding of the glenoidal reattachment of the IGHL into a hammock-like position around the humeral head, were assumed (Fig. 1d). According to the standards of open surgery, the finger probe between the capsule and humeral head was negative and aimed for a distance of 5 mm.

**Fig. 2:**
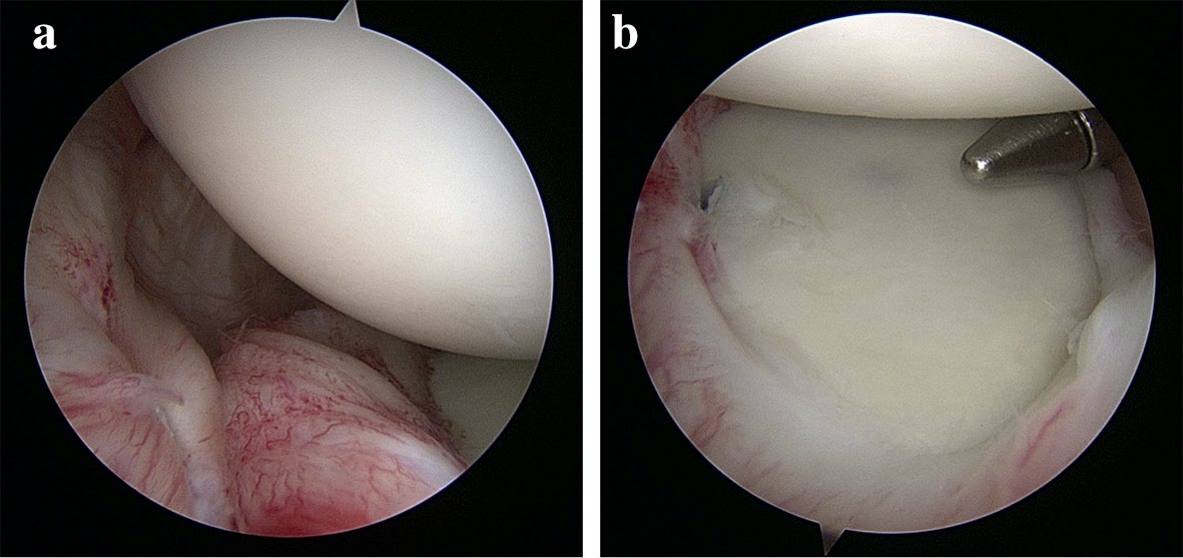
Arthroscopic Findings Following Arthroscopic Bankart Repair with an Individualized Capsular Shift. Right shoulder in lateral decubitus position (view from anterior–superior portal). **a** Anatomic anterior-inferior capsulolabral reconstruction after completing arthroscopic Bankart repair with an individualized capsular shift using two suture anchors at the 5.00 and 3.30 positions. **b** Re-centred location of the humeral head within the glenoid cavity

Next, a double-loaded 3.0-mm Bio-FASTak anchor (Arthrex, Naples, FL) was implanted at the 3.30 position. As individually required, one or two FiberWire #2 mattress stitches were used for refixation of the anterior capsulolabral complex from the 2.00 position to the 3.30 position applying the same principles. The most superior stitch incorporated the glenoidal insertion site of the medial glenohumeral ligament (MGHL). We reinserted and did not shorten or shift, the MGHL to prevent restriction of external rotation. The intermediate and final results of the capsulolabral reconstructions were continuously controlled from the anterior–superior viewing portal. Efficient capsulolabral reconstruction was assumed in the case of perfect centring of the humeral head within the glenoid cavity, the presence of a labral "bump" and the complete unfolding of the anterior-inferior capsular pouch into a hammock-like position around the humeral head (Fig. 2a/b).

### Capsular volume measurement

All shoulder arthroscopies were performed with an isotonic saline solution using an arthroscopic pump with standardized settings (pressure 50 mmHg, flow 0.75 L/min). Pre-intervention volumetric measurements of the capsular volume were performed prior to cannula placement in the instability group as well as in the control group. The physicians performing the preoperative clinical examinations and deciding on the inclusion of patients in the study did not perform capsular volume measurements. All involved surgeons were blinded to laxity and clinical histories (date and mechanism of trauma). After diagnostic glenohumeral arthroscopy, the inflow was closed, and the fluid was immediately aspirated from the joint under arthroscopic control. Fluid aspiration was performed via the posterior portal using a 50.0-mL perfusor-syringe with a watertight luer-lock attachment to the outflow of the arthroscope.

The measurements were repeated three times under arthroscopic control, and the mean value was calculated. Likewise, post-interventional measurements in the instability group were performed with two inset cannulas (Twist-In™-Cannula, 8.25 mm × 9 cm anterior-inferior, Crystal Cannula®, 5.75 mm × 7 cm anterior–superior, Arthrex, Naples, FL). The implantation depths of the cannulas were standardized in terms of two visible threads inside the joint. The volume difference between the filling volume of the cannula and the volume displaced by the cannula itself was calculated based on a water bath experiment. This 1.5-mL difference was added to the post-interventional joint volume measurements. A pilot study by Lubiatkowski et al. showed that in vivo intraoperative measurement of the shoulder joint volume is a very reliable method with high intraclass correlation coefficients (ICCs), which range from 0.996 to 0.997 [21].

### Statistical analysis

The statistical analyses and presentation of the data were completed using the software IBM SPSS Statistics for Macintosh version 24.0 (IBM Corp. in New York, USA). Continuous variables were reported as the mean with one standard deviation. Differences between groups of data with a normal distribution were analysed with Student’s t-test. If data showed non-normal distributions based on the Shapiro–Wilk test, the non-parametric Kruskal–Wallis test and Mann–Whitney *U*-test were used. The level of significance was set at *P* < 0.05. Intra-class correlation coefficients (ICCs) were calculated for pre- and post-interventional volume measurements to prove reliability. The relative capsular volume reduction (%) was calculated by dividing the difference between the pre- and post-interventional volume by the pre-interventional joint volume and multiplying by 100. The Pearson coefficient (*r*) was used to evaluate the correlation between the capsular volume and the biometric data of the control group (sex, age, height, weight and BMI). Linear regression was performed to describe the correlations.

A priori power analysis revealed that 28 patients would be needed in each group (instability and control) to detect a volumetric difference of 4.0 mL (± 5.0 mL) at a significance level of *P* = 0.05 with a power of 90%. According to Lubiatkowski et al. [21], 7 patients per subgroup would be required to detect a volumetric difference of 4.0 mL (± 10.0 mL) between hyperlax and non-hyperlax patients.

## Results

### Study population

Between 2013 and 2017, a total of 82 patients were included in the study. The control group consisted of 50 patients (27 female, 23 male; average age, 50.6 ± 7.8 years; range, 27–62 years). The instability group included 32 patients (4 female, 28 male) and underwent intraoperative measurements of shoulder joint volumes before and after arthroscopic Bankart repair with an individualized capsular shift. The mean age of the instability patients at surgery was 25.8 ± 6.8 years (range 15–47 years). Fifteen patients did not show any clinical signs of ligamentous hyperlaxity, while 17 had hyperlaxity. High intra-class correlation coefficients (ICCs) ranging from 0.959 (postinterventional volume) to 0.998 (pre-interventional volume) were achieved.

### Control group

Significant sex-specific differences were found for height (*P* < 0.01), weight (*P* < 0.01), BMI (*P* = 0.01) and capsular volume (*P* < 0.01), with consistently lower values for female patients (height 166.3 ± 5.4 cm vs. 179.3 ± 6.5 cm; weight 67.1 ± 11.9 kg vs. 88.9 ± 15.8 kg; BMI 24.2 ± 4.1 kg/m^2^ vs. 27.5 ± 4.0 kg/m^2^; capsular volume 17.4 ± 4.0 mL vs. 25.4 ± 7.2 mL). Based on the control group data, reference values for physiological shoulder joint volumes were calculated and correlated with biometric parameters (sex, age, height, weight and BMI). Significant Pearson correlations were found for sex, size and weight, as shown in Table 1.

**Table 1:** Correlation of biometric and volumetric parameters (Control Group)

	Pearson correlation coefficient	*P* value
Sex	− 0.58	< 0.01*
Age	− 0.18	n.s
Height	0.46	0.01*
Weight	0.39	0.01*
BMI	0.24	n.s

**Fig. 3:**
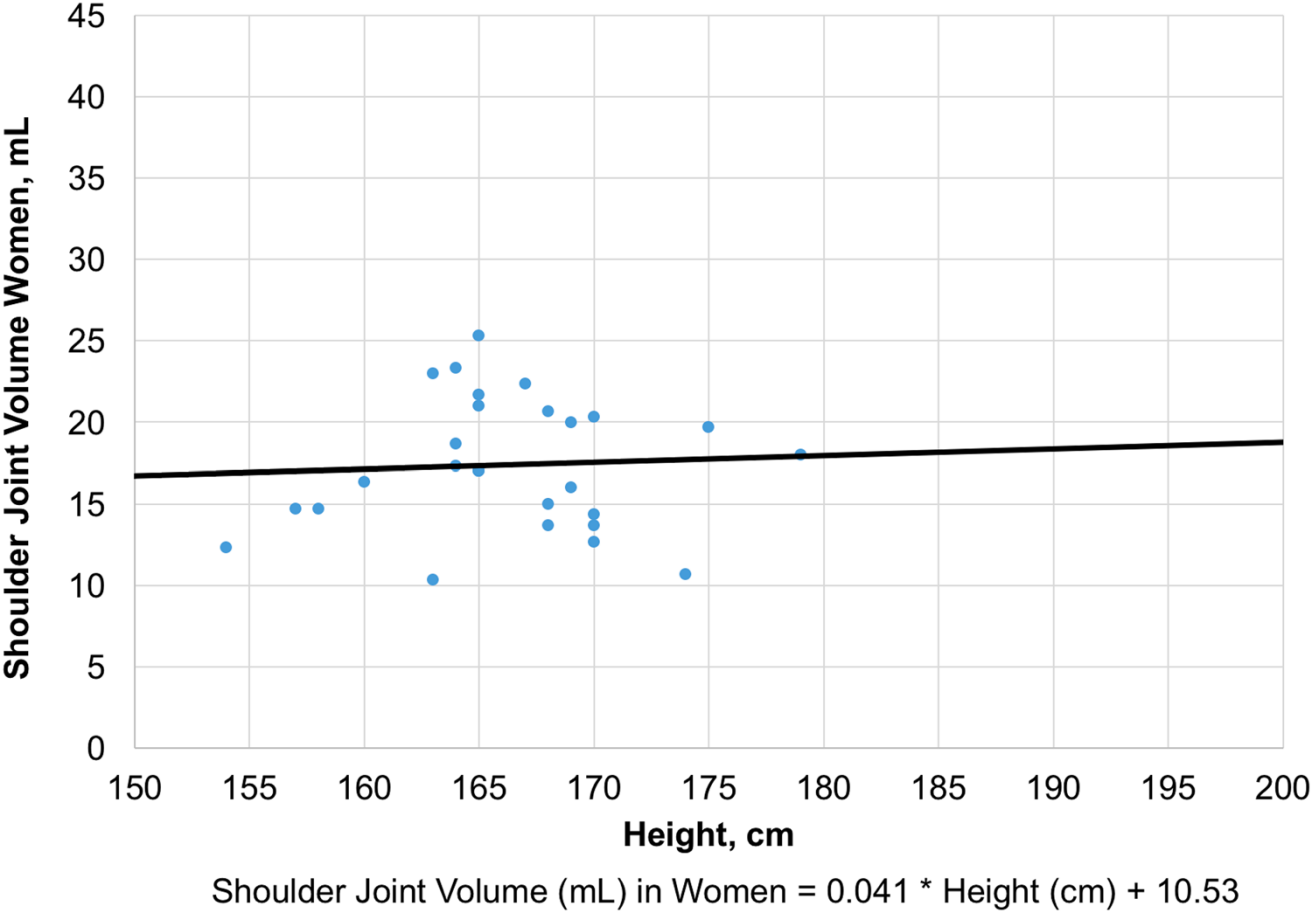
Calculation of Physiological Shoulder Joint Volume in Women based on the Control Group

**Fig. 4:**
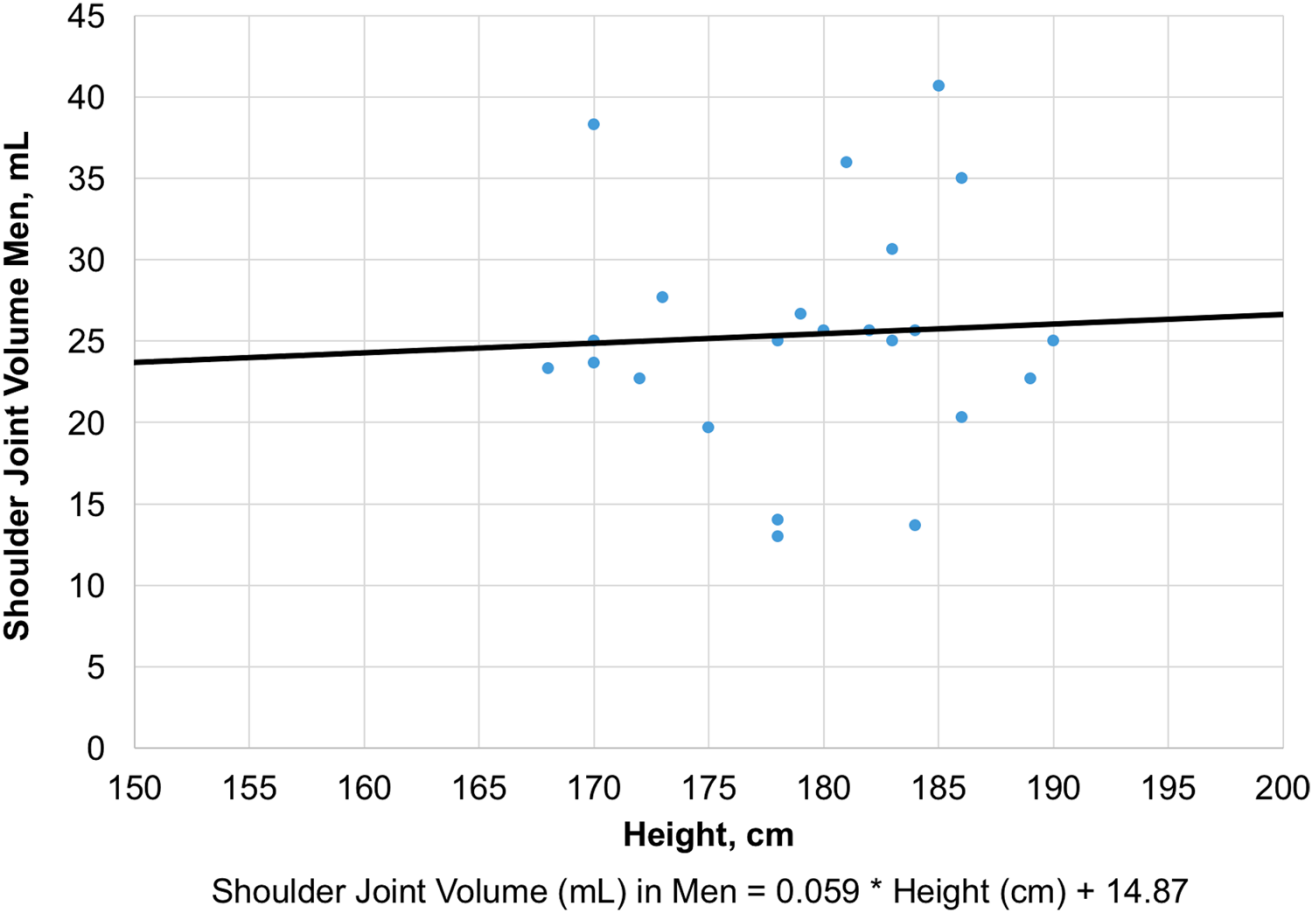
Calculation of Physiological Shoulder Joint Volume in Men based on the Control Group

Using a linear regression model, these parameters were used to estimate individual physiological shoulder joint volumes. The biometric parameters sex, height and weight (*R*-squared = 0.58) were included in the most accurate estimation. The following sex-specific formula can be used for calculating individual physiological shoulder joint volumes: Shoulder joint volume (mL) in women = 0.03 * height (cm) + 0.02 * weight (kg) + 12.0; Shoulder joint volume (mL) in men = 0.06 * height (cm)  − 0.004 * weight (kg) + 14.2. A less accurate estimation (*R*-squared = 0.21) can be derived from sex-specific interpolation diagrams based on the parameter height (Figs. 3 and 4).

### Instability group

The pre-interventional volume was significantly higher in instability patients with ligamentous hyperlaxity than in non-hyperlax patients (30.8 mL ± 5.9 vs. 39.9 mL ± 11.9; *P* = 0.02) (Table 2). Arthroscopic Bankart repair with an individualized capsular shift reduced shoulder joint volumes by a mean of 51.0% (± 20.8) in hyperlax patients and 38.4% (± 16.1) in non-hyperlax patients (*P* = 0.03). Comparable numbers of capsulolabral sutures were used in the hyperlax subgroup and non-hyperlax subgroup (6.9 ± 2.1 vs. 6.7 ± 1.7; *n.s.*). Thus, each capsulolabral suture reduced the capsular volume by 7.4 ± 8.3% and 5.8 ± 3.7% (*n.s.*), respectively. Comparable post-interventional capsular volumes were achieved in both subgroups (19.5 ± 5.1 mL and 19.0 ± 5.7 mL; *n.s.*). Apart from patient height, there were no significant sex-specific differences related to biometric parameters.

**Table 2:** Biometric and volumetric data (Instability Group)

	All instability patients	Instability, no hyperlaxity	Instability, hyperlaxity	*P* Value
Sex, female/male, *n*	4/28	2/13	2/15	n.s
Age, years	25.8 ± 6.8	26.4 ± 6.9	25.1 ± 6.8	n.s
Height, cm	180.6 ± 7.5	178.6 ± 5.9	182.4 ± 8.5	n.s
Weight, kg	81.2 ± 15.1	77.3 ± 12.5	84.6 ± 16.6	n.s
BMI, kg/m^2^	24.7 ± 3.4	24.2 ± 3.6	25.2 ± 3.3	n.s
Pre-interventional volume, mL	35.6 ± 10.5	30.8 ± 5.9	39.9 ± 11.9	0.02*
Post-interventional volume, mL	19.3 ± 5.4	19.0 ± 5.7	19.5 ± 5.1	n.s
Absolute volume reduction, mL	16.3 ± 7.8	11.8 ± 5.0	20.3 ± 8.3	0.03*
Relative volume reduction, %	45.9 ± 21.9	38.4 ± 16.1	51.0 ± 20.8	0.03*
Capsulolabral sutures, *n*	6.8 ± 1.9	6.7 ± 1.7	6.9 ± 2.1	n.s
Absolute volume reduction per suture, mL	2.4 ± 1.7	1.8 ± 1.2	3.0 ± 3.3	n.s
Relative volume reduction per suture, %	6.8 ± 4.8	5.8 ± 3.7	7.4 ± 8.3	n.s

### Instability group vs. control group

**Fig. 5:**
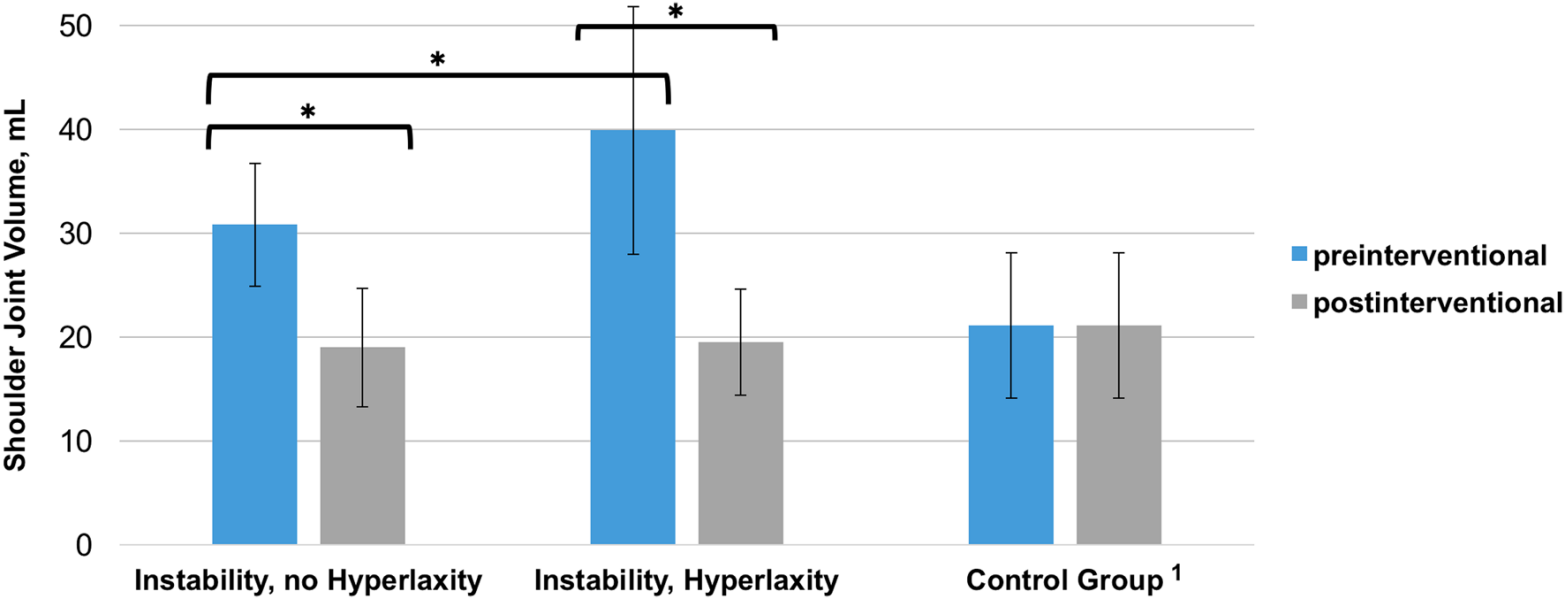
Volume Measurements. Post-interventional volumes equal pre-interventional volumes in the control group

Significant differences between instability patients and the control group were found related to the biometric parameters sex, age and height (all *P* < 0.01). The mean pre-interventional capsular volume was significantly higher in the instability group than in the control group (35.6 ± 10.5 mL vs. 21.1 ± 7.0 mL; *P* < 0.01). Following arthroscopic Bankart repair with an individualized capsular shift, both groups showed comparable shoulder joint volumes without significant differences (19.3 ± 5.4 mL vs. 21.1 ± 7.0 mL; *n.s.*). Compared to the control group data, physiological post-interventional shoulder joint volumes were achieved for all instability patients and for the two subgroups of hyperlax and non-hyperlax patients (Table 3 and Fig. 5).

**Table 3:** Comparison of Biometric and Volumetric Data (Instability vs. Control Group)

	Instability group	Control group	*P* value
Sex, female/male, *n*	4/28	27/23	< 0.01*
Age, years	25.8 ± 6.8	50.6 ± 7.8	< 0.01*
Height, cm	180.6 ± 7.5	172.3 ± 8.8	< 0.01*
Weight, kg	81.2 ± 15.1	77.1 ± 17.5	n.s
BMI, kg/m^2^	24.7 ± 3.4	25.8 ± 4.3	n.s
Pre-interventional volume, mL	35.6 ± 10.5	21.1 ± 7.0	< 0.01*
Post-interventional volume, mL	19.3 ± 5.4	21.1 ± 7.0^a^	n.s

^a^Post-interventional volumes equal pre-interventional volumes in the control group

## Discussion

The most important finding of the present study was that arthroscopic Bankart repair with an individualized capsular shift enabled the restoration of physiological shoulder joint volumes in hyperlax and non-hyperlax patients with anterior shoulder instability. More specifically, Arthroscopic Bankart repair with an individualized capsular shift resulted in a mean capsular volume reduction of 45.9% employing 6.8 capsulolabral sutures on average. A significantly larger capsular shift (+ 12.6%) was performed in hyperlax patients than in non-hyperlax patients. However, the number of employed capsulolabral sutures did not differ between the two subgroups.

Both imaging studies and surgical in vivo studies found pathological capsular enlargements of unstable shoulder joints [9, 10, 14, 21]. Dewing et al. [9] demonstrated an increased cross-sectional area of the capsule in patients with posterior or multidirectional instability versus controls, as measured by magnetic resonance arthrography [9]. Park et al. [30] investigated time-dependent changes in capsular volumes in patients undergoing arthroscopic Bankart repair and capsular shift by means of computed tomography arthrography. The mean capsular volume significantly decreased from 20.1 mL to 14.8 mL 3 months after surgery but then significantly increased to 18 mL one year postoperatively. This recurrence was attributed to the viscoelasticity of capsuloligamentous structures.

The present study clearly showed that the pre-interventional capsular volumes were significantly higher in patients with anterior shoulder instability than in controls (35.6 ± 10.5 mL vs. 21.1 mL ± 7.0, *P* < 0.01). These findings are consistent with the in vivo study of Lubiatkowski et al. [21], who employed a comparable experimental protocol. In their study, shoulder joint volumes were significantly higher in patients with anterior shoulder instability than in a control group of patients with subacromial impingement syndrome (38.9 mL vs. 25.6 mL, *P* < 0.01) [21]. Compared to our findings, slightly higher capsular volumes in both instability patients and controls using an almost twice as high pump pressure (90 mmHg) were measured by the authors. The brand and type of the pump used by Lubiatkowski et al. were not specified. The use of a different pump might entail a variation in absolute measurements. However, this potential systematic deviation did not impair the validity of this study. However, they found an almost identical volumetric difference. We put special emphasis on the formation of a representative and a conclusive control group consisting of patients with physiological capsular patterns and unrestricted ranges of shoulder motion. Contrary to Lubiatkowski et al. [21], we found significantly higher pre-interventional capsular volumes in hyperlax (+ 30%) patients compared to non-hyperlax patients (39.9 ± 11.9 mL vs. 30.8 ± 5.9 mL; *P* = 0.02). In this context, the present in vivo study proves and quantifies the pathogenetic importance of ligamentous hyperlaxity in anterior shoulder instability.

Effective soft tissue stabilization requires both a biomechanically stable Bankart (labral) repair and adequate soft-tissue rebalancing by means of an individually adjusted capsular shift [1, 34, 36, 37]. In 1980, Neer and Foster described the inferior capsular shift as an effective open procedure for the treatment of capsular and ligamentous redundancy in involuntary inferior and multidirectional shoulder instability [27]. Lubowitz et al. [23] quantified the capsular volume reductions of Neer's open capsular shift in a cadaveric model using three different techniques of volume measurement: MRI, ultrasound, direct needle injection and aspiration. On average, capsular volumes were found to be significantly reduced by 57%, 60% and 54%, respectively. Subsequently, arthroscopic techniques for capsular shifts and plications evolved, but cadaveric models showed inferior efficacies of capsular volume reduction compared to conventional open procedures [2, 5, 7, 11, 13, 16, 17, 22–25, 33, 38, 39]. In an in vitro study, Cohen et al. [7] reported a capsular volume reduction of 22.8% employing three arthroscopic 1-cm capsular shifts at the 3.00, 5.00 and 8.00 positions. Other cadaveric studies reported relative capsular volume reductions ranging from 16–34% [11, 17]. In the present in vivo study, arthroscopic Bankart repair with an individualized capsular shift effectuated a mean capsular volume reduction of 46%, with significant differences between non-hyperlax and hyperlax patients (38.4% vs. 51%). The present study shows that modern arthroscopic techniques for capsular volume reconstruction may be as effective as previous open techniques [7, 11, 17, 23–25, 39]. In view of a long-term recurrence rate of up to 30% after arthroscopic Bankart repair, open stabilization may be the most efficient and safe procedure, especially in patients with multidirectional instability [3].

The in vivo study of Lubiatkowski et al. [21] that reported a 37% capsular volume reduction after arthroscopic labral repair and a 61% capsular volume reduction after arthroscopic capsular shift on average was consistent with our findings. However, this study group did not specify the number of labral and capsular sutures. The first in vivo study quantifying both absolute capsular volume reduction and relative contributions per capsulolabral suture is presented herein. For the restoration of physiological shoulder joint volumes, a mean of 6.8 capsulolabral sutures was applied. Each stitch effectuated a mean absolute and relative capsular volume reduction of 2.4 mL and 6.8%, respectively. Capsular volume reduction in hyperlax patients exceeded that in non-hyperlax patients by 8.5 mL (12.6%). However, the mean number of capsulolabral sutures did not differ between the groups (Table 2).

To date, arthroscopic shoulder surgeons have not had at their disposal reliable tools to control the individually required extent of capsular volume reduction. Furthermore, normative capsular volumes could not be determined individually. Throughout the literature, the range of physiological glenohumeral volumes varies widely between 10 and 38 mL. The most likely reasons for this are differences with regard to experimental volume measurements, study populations and coexisting pathologies [10, 21, 23, 28]. Control group patients showed neither coexisting pathologies (other than calcific tendinitis), nor clinical signs of pathological capsular patterns. Therefore, the value of 21 mL may be regarded as the mean physiological shoulder joint volume in our experimental setting. In accordance with the study of Dietz et al. [10], we found highly significant correlations with sex, height and weight. Based on the control group data analysis, a sex-specific formula allowing for an accurate calculation of individual, physiological shoulder joint volumes was derived (Figs. 3, 4).

The present study has certain limitations. Although the employed method of capsular volume measurement has been proven to be reliable, the occurrence of measurement errors could not be fully excluded. However, we performed three consecutive measurements, which were highly reliable, and their mean value was used for the statistical analysis to minimize accidental measurement errors. Periarticular soft tissue swelling may have had a reducing influence on post-interventional volume measurements. This systematic effect equally affected all patients receiving arthroscopic Bankart repair with an individualized capsular shift and was controlled for by a consistent use of cannulas directly after completion of the post-interventional volume measurements. Since there exists no direct communication between the subscapular and subcoracoid bursa and the joint cavity, relevant fluid escape into the bursae should not have occurred [8]. The size of the foramen of Weitbrecht is not known to correlate with ligamentous hyperlaxity. Thus, individual anatomical variations should not have systematically influenced the results of this study.

An individualized technique of arthroscopic capsulolabral reconstruction in anterior shoulder instability is presented. Hereby, over- or under correction of the capsular volume could be effectively avoided. In future clinical routine, the individual target volume could be determined preoperatively. Intraoperative comparison with the pre-interventional pathological volume allows for control of the individually adjusted capsular shift (capsular volume reduction). However, prospective studies are required to assess the clinical impacts of this individualized approach. Moreover, evolution of capsular volume reduction should be monitored over time.

## Conclusions

Hyperlax and non-hyperlax instability patients exhibited higher shoulder joint volumes than non-hyperlax patients in the control group. Arthroscopic Bankart repair with an individualized capsular shift restored physiological capsular volumes in hyperlax and non-hyperlax patients with anterior shoulder instability. The biometric factors sex, body height and weight significantly influenced physiological shoulder joint volumes. Current findings allow for individual adjustment and intraoperative control of the capsular shift to avoid over- or under correction of the capsular volume.
