# Correction to: Arthroscopic Bankart repair with an individualized capsular shift restores physiological capsular volume in patients with anterior shoulder instability

**DOI:** 10.1007/s00167-021-06669-7

**Published:** 2021-08-02

**Authors:** Helge Eberbach, Martin Jaeger, Lisa Bode, Kaywan Izadpanah, Andreas Hupperich, Peter Ogon, Norbert P. Südkamp, Dirk Maier

**Affiliations:** 1 Department of Orthopaedic and Trauma Surgery, Medical Center‑University of Freiburg, Faculty of Medicine, University of Freiburg, Hugstetter Straße 55, 79106 Freiburg, Germany; 2 Center of Orthopaedic Sports Medicine, Breisacher Str. 84, 79110 Freiburg, Germany

## Correction to: Knee Surgery, Sports Traumatology, Arthroscopy (2021) 29:230–239 10.1007/s00167-020-05952-3

The article “Arthroscopic Bankart repair with an individualized capsular shift restores physiological capsular volume in patients with anterior shoulder instability”, written by Helge Eberbach, Martin Jaeger, Lisa Bode, Kaywan Izadpanah, Andreas Hupperich, Peter Ogon, Norbert P. Südkamp and Dirk Maier, was originally published Online First without Open Access. After publication in volume 29, issue 1, page 230–239 the author decided to opt for Open Choice and to make the article an Open Access publication. Therefore, the copyright of the article has been changed to © The Author(s) 2021 and the article is forthwith distributed under the terms of the Creative Commons Attribution 4.0 International License, which permits use, sharing, adaptation, distribution and reproduction in any medium or format, as long as you give appropriate credit to the original author(s) and the source, provide a link to the Creative Commons licence, and indicate if changes were made. The images or other third party material in this article are included in the article’s Creative Commons licence, unless indicated otherwise in a credit line to the material. If material is not included in the article’s Creative Commons licence and your intended use is not permitted by statutory regulation or exceeds the permitted use, you will need to obtain permission directly from the copyright holder. To view a copy of this licence, visit http://creativecommons.org/licenses/by/4.0/. Open access funding enabled and organized by Projekt DEAL.

The original article has been corrected.

